# A case report of childhood cutaneous cartilaginous tumor located in the neck

**DOI:** 10.3389/fped.2025.1607480

**Published:** 2025-10-10

**Authors:** Yijia He, Zhuochen Wu, Qing Zhu, Jinxu Qi, Xiaomei Xuan, Guoqiang Zhang

**Affiliations:** ^1^ Department of Dermatology, The First Hospital of Hebei Medical University, Shijiazhuang, Hebei, China; ^2^ Hebei Technical Innovation Center for Dermatology and Medical Cosmetology Technology, Shijiazhuang, Hebei, China; ^3^ Subcenter of National Clinical Research Center for Skin and Immune Diseases, Shijiazhuang, Hebei, China

**Keywords:** cutaneous cartilaginous tumor, chondromacutis, children, anterior neck, benign skin tumour

## Abstract

**Background:**

Cutaneous Cartilaginous Tumor, also known as Chondromacutis, is a rare benign tumour of the skin that develops in the hands and feet. It is extremely rare to find lesions located in the neck. It typically occurs in adults aged 30–60 years and is rare in children, with no gender predilection. The disease may have a mesenchymal cellular origin. The tumour is located in the dermis and may involve subcutaneous tissue.

**Case:**

In this paper, we report a case of a 6-year-old child. The child was born with a skin-coloured hemispherical mass with a smooth surface on the right side of her neck. The swelling gradually increased in size and she presented to the dermatology department. Histopathological examination confirmed Cutaneous Cartilaginous Tumor.

**Conclusion:**

Reports of Cutaneous Cartilaginous Tumor located in the neck of children are extremely rare. The case of Cutaneous Cartilaginous Tumor in the right neck of a 6-year-old child reported in this study supplements the clinical data of this tumor in special populations and special locations, provides a typical case reference for clinical identification of such rare benign tumors, and also accumulates basic data for existing literature and subsequent related research.

## Introduction

Cutaneous Cartilaginous Tumor also known as Chondromacutis is a rare benign tumour of the skin. Its pathogenesis is unclear and may be related to genetic mutations and inheritance. The tumour is mostly located on the fingers, toes and soles of the feet. It is clinically rare and accounts for approximately 1.5% of all cutaneous chondromas (1, 2). It can occur at any age. Reports of Cutaneous Cartilaginous Tumor of the neck in children are extremely rare, therefore, we report a case of Cutaneous Cartilaginous Tumor of the anterior neck in a child, aiming to enhance the understanding of and attention to this rare disease.

## Case presentation

**Figure 1:**
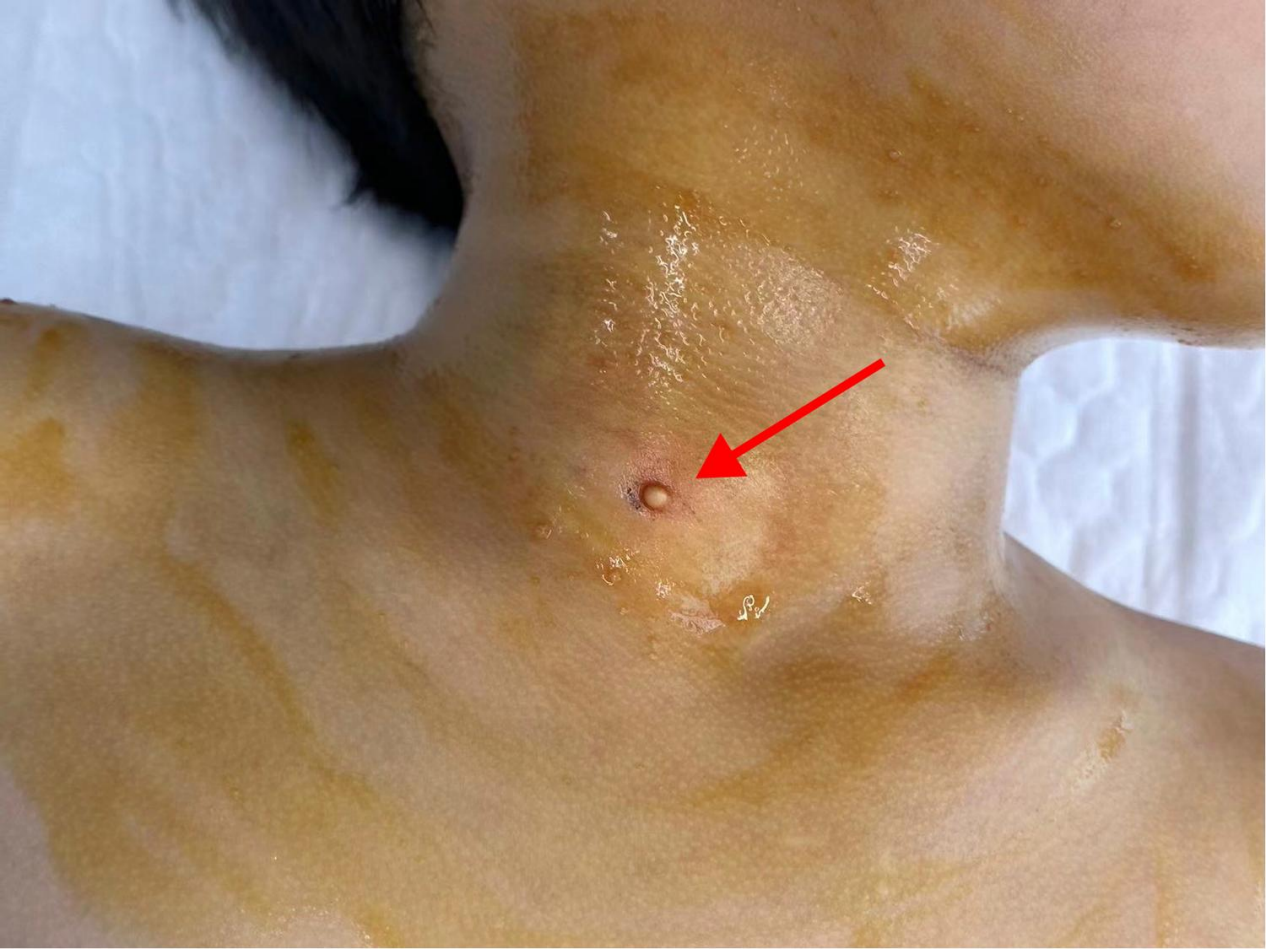
A well-defined, skin-colored growth is present on the right side of the neck. It has a hemispherical shape, a smooth surface, and an approximate diameter of 0.5 cm, is elevated above the skin surface, and is well demarcated from the surrounding normal skin.

**Figure 2:**
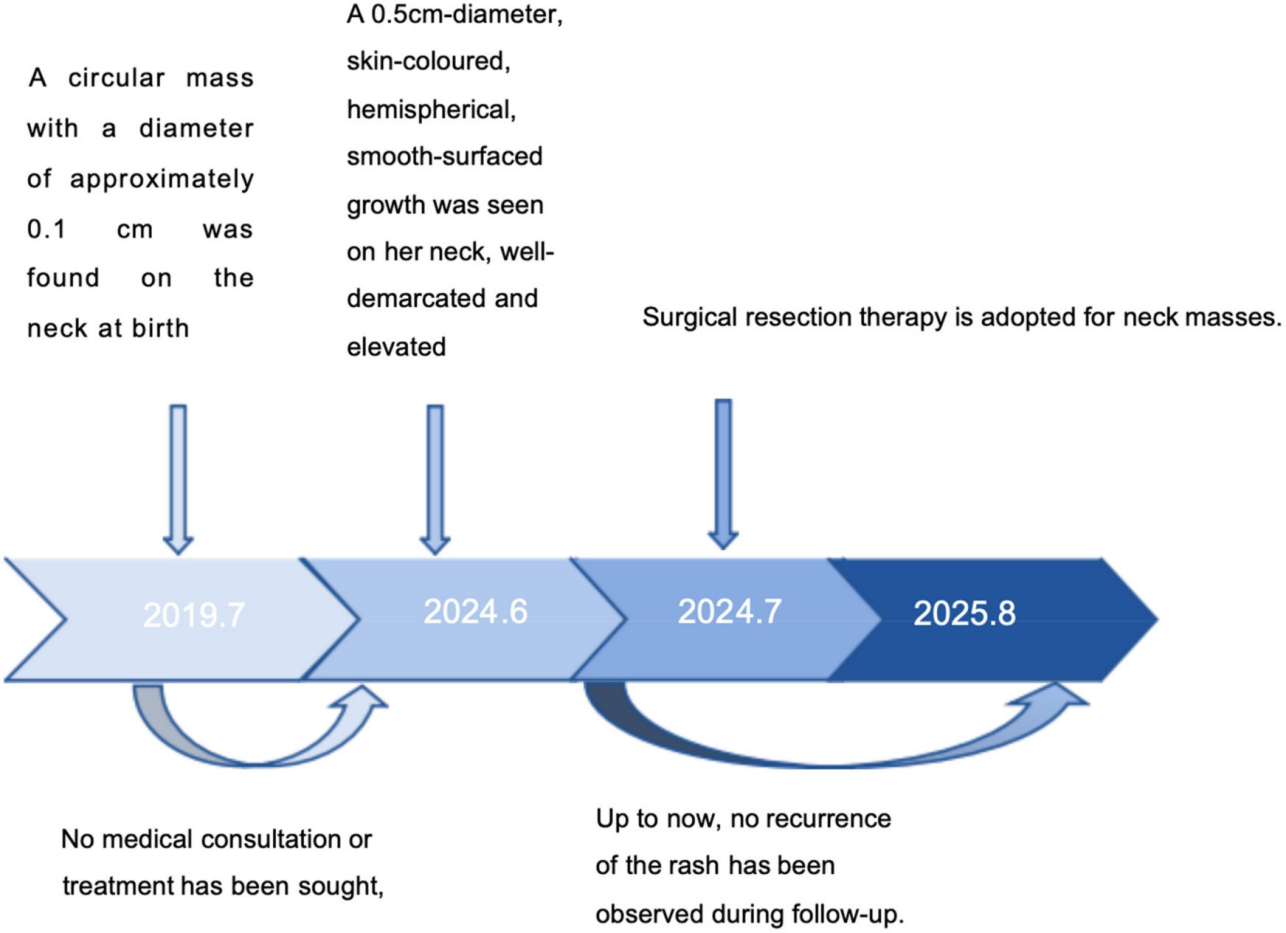
Timeline illustrating the key stages and chronological progression of the patient's disease course.

**Figure 3:**
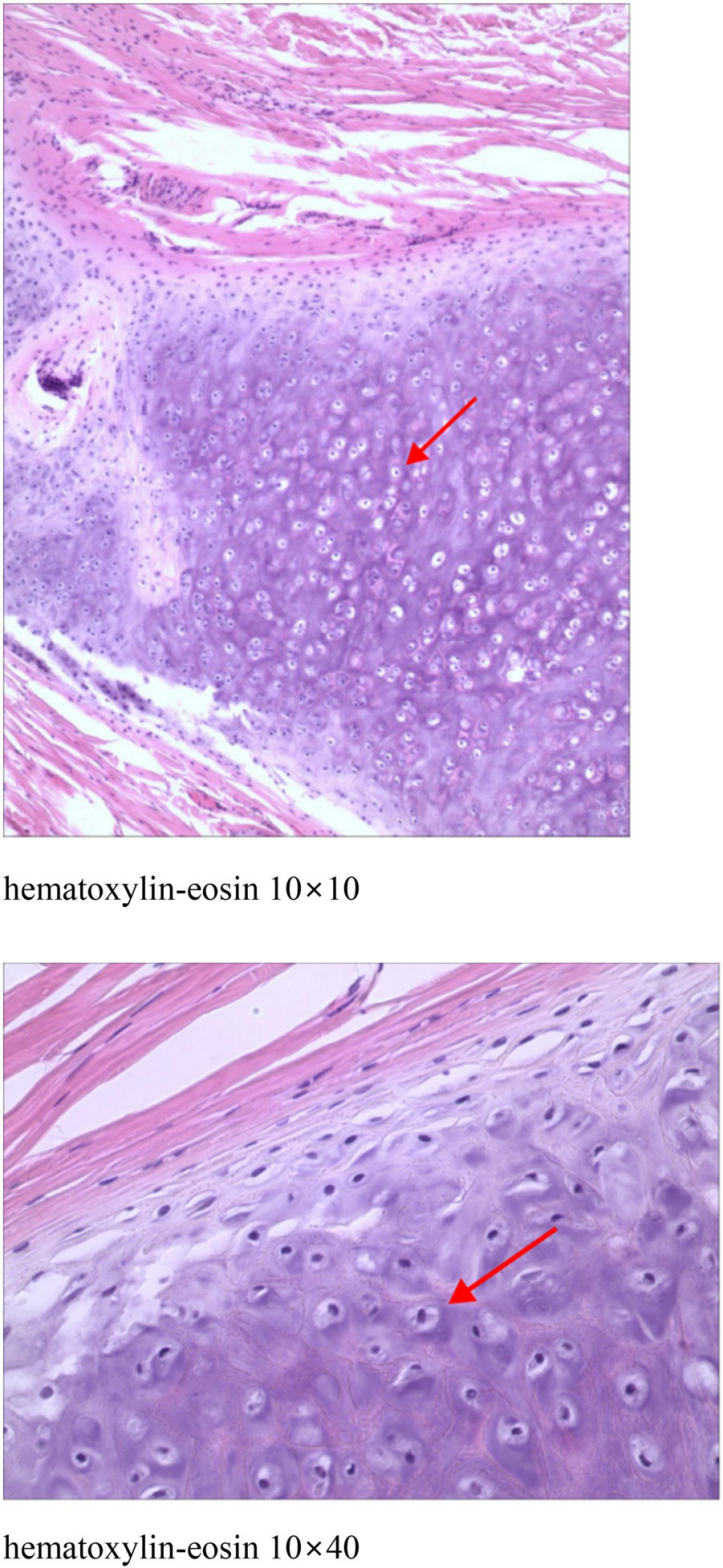
Epidermis showed irregular hyperplasia with intact basal cells and coarse reddish staining of dermal collagen fibres. A nodular structure was seen within the subcutaneous fat layer, which had a fibrous pseudo-envelope on the outer side and was composed of hyaline chondrocytes on the inner side, and dilated capillaries were seen.

The patient was a 6-year-old female child. She was found at birth with a skin-coloured hemispherical swelling on the right side of her neck with a smooth surface. The swelling gradually increased in size and she presented to the dermatology department. She was otherwise healthy and had no relevant family history. On examination, a diameter approximately 0.5 cm skin-coloured growth with a hemispherical shape and a smooth surface was seen on her neck, which was well demarcated from the surrounding normal skin, elevated above the skin surface, as shown in Figure 1 (for details of the timeline, see Figure 2). Based on the patient's clinical manifestations, the diseases we consider include cutaneous chondroma, sebaceous cyst, calcifying epithelioma, and so on. Local ultrasound revealed: a subcutaneous round nodule in the neck with clear boundaries, showing heterogeneous hypoechoic areas and scattered hyperechoic foci within it; color Doppler flow imaging indicated no obvious blood flow signals. We excised this growth completely by designing an elliptical incision, performing blunt dissection in the loose connective tissue plane between the subcutaneous fat and superficial fascia, resecting the tumor capsule entirely, and using a tension-reducing suture technique, and performed histopathological examination. Microscopically, it could be seen that the epidermis showed irregular hyperplasia with intact basal cells and coarse reddish staining of dermal collagen fibres. A nodular structure was seen within the subcutaneous fat layer, which had a fibrous pseudo-envelope on the outer side and was composed of hyaline chondrocytes on the inner side, and dilated capillaries were seen, as shown in Figure 3. Due to economic considerations, the patient did not undergo immunohistochemical examination. The patient was diagnosed with Cutaneous Cartilaginous Tumor. We followed the patient till now and no recurrence was found.

## Discussion

Chondromas can be classified as exophytic chondromas, endophytic chondromas, and periosteal chondromas. Exophytic chondrosarcoma, also known as soft tissue chondrosarcoma or extraosseous chondrosarcoma, occurs in soft tissues unrelated to bone and periosteum (3). Exophytic chondrosarcoma is a rare benign cartilage tumour that occurs in more than 80% of the fingers, followed by the feet. Chondromas of the dura mater, larynx, pharynx, oral cavity, parotid glands are less common and are extremely rare in the neck (1, 4). Cutaneous Cartilaginous Tumors include chondroma cutis (CC) arising from the dermis and soft tissue chondromas that may involve the subcutis; some literature groups these two under “extraskeletal chondromas” due to overlapping features (5). Their morphology consists mainly of hyaline cartilage mixed with mucus-like stroma (6). They occur between the ages of 30–60 years, with no difference between men and women, and are rare in children (4, 7). In clinical practice, cutaneous cartilage tumour is a benign cartilage tumour that occurs in more than 80% of cases in the fingers, followed by the feet (8).

Clinically, Cutaneous Cartilaginous Tumor is often asymptomatic. It is usually an unintentionally detected painless mass or isolated nodule that is slow growing, usually <3 cm in diameter, well defined, hard, and cartilaginous in histological section (1).

The pathogenesis of the disease is unclear and is currently thought to be related to two factors: 1. residual embryonic tissue within the cartilaginous region of the pre-existing fetus. 2. Stimulation by some factor, such as trauma (1). In children, congenital Cutaneous Cartilaginous Tumor presents as an asymptomatic, painless, and progressive neck swelling. In contrast, adults with acquired Cutaneous Cartilaginous Tumor often manifest clinical symptoms, such as local pain and nerve compression, which may be associated with local compression effects that develop following prolonged progression of the tumor. However, this observation is based on limited case data, and this hypothesis needs to be confirmed by further increasing the sample size.

Imaging is helpful in the diagnosis of Cutaneous Cartilaginous Tumor in the neck. Ultrasound can be used to determine whether the nature of the nodule is substantial or otherwise. On CT, Cutaneous Cartilaginous Tumor presents as a well-defined medium to high density mass with varying degrees of calcification (1, 9). On MRI, it often presents as low to moderate signal on T1-weighted imaging and high signal on T2-weighted imaging.Histopathology is key for diagnosis: it shows well-circumscribed, lobulated nodules in the dermis surrounded by a fibrous capsule, composed of mature hyaline cartilage, occasionally with ossification/calcification and no abnormal mitosis (5, 10, 11). Additionally, immunohistochemistry can identify the chondrocyte differentiation property of tumor cells by detecting specific protein markers, thereby assisting in confirming the tumor type and ruling out other similar tumors.

The differential diagnosis includes highly differentiated extraosseous chondrosarcoma, extraosseous mucinous-like chondrosarcoma, mesenchymal chondrosarcoma, Congenital Dermatofibrosarcoma Protuberans (CDFP), Dermoid cyst, Thyroglossal ductcyst, Accessory tragus, congenital cartilaginous rests of the neck,Cervical chondrocutaneous remnant or branchial anomalies, Choristoma. Highly differentiated extraosseous chondrosarcoma exhibits abnormal mitoses, heterogeneity, and necrosis. Extraosseous mucinous-like chondrosarcoma is less differentiated, especially in the peripheral part of the tumour. Mesenchymal chondrosarcoma is another chondroid-like lesion with a characteristic dimorphic pattern consisting of well-differentiated cartilage surrounded by small, undifferentiated tumour cells (7, 12). Congenital DFSP features the specific COL1A1-PDGFB fusion gene, with spindle cells in a storiform arrangement and positive CD34 under the microscope; cutaneous chondroma has no such genetic abnormality, showing mature cartilage tissue in histopathology (13). Dermoid cysts occur mostly in embryonic development-abnormal areas (e.g., head and face) with cyst walls containing skin appendages (e.g., hair, sebaceous glands) and oil-like contents, while cutaneous chondroma has no cyst wall and is pathologically characterized by mature cartilage tissue (differentiable via pathological examination). Thyroglossal duct cysts locate near the anterior neck midline, relate closely to the hyoid bone (moving with swallowing/tongue protrusion), and have stratified squamous epithelial cyst walls without cartilage—distinct from cutaneous chondroma's subcutaneous hard nodules and cartilage-related pathology (14). Accessory tragi, mostly congenital, are cartilage-containing skin protrusions near the tragus/ear (similar to normal tragus in appearance); cutaneous chondroma occurs anywhere (no typical periauricular distribution) and lacks such protrusions, differentiated by onset site and appearance (15). Congenital cartilaginous rests of the neck are typically located along the anterior border of the sternocleidomastoid muscle in the neck, presenting as firm, painless masses. Histopathologically, they show elastic cartilage enclosed by various skin structures like eccrine glands, adipose tissue, and pilosebaceous units, which is distinct from the mature cartilage tissue in cutaneous chondroma. Cervical chondrocutaneous remnant or branchial anomalies usually embed in the anterior border of the sternocleidomastoid muscle, often appearing as skin tags. These anomalies likely originate from the second branchial arch and can be differentiated from cutaneous chondroma by their appearance and embryological origin. Choristoma is an ectopic mass of normal tissue, and its histological composition depends on the type of tissue involved. It lacks the characteristic mature cartilage seen in cutaneous chondroma, and its occurrence in unusual locations further aids in the differential diagnosis (16–18). In the present case report, the histopathological manifestation of the nodule consisted of hyaline chondrocyte-like cells, and no anisotropy or other manifestations of chondrosarcoma were found.

Surgical resection is currently the treatment of choice in all cases (19), with a recurrence rate of approximately 10%–18% and progression to chondrosarcoma in some cases after recurrence (20). Chung and Enzinger reported a case of recurrence at the 38th year postoperatively in a patient who had a long follow-up period (21). The present patient has been followed up for a period of 2 months now, and has not shown any recurrence.

## Conclusion

Reports of Cutaneous Cartilaginous Tumor of the neck in children are very rare up to now. This paper has the limitation of having too few cases. This report highlights the importance of considering cutaneous cartilaginous tumours in the differential diagnosis of congenital neck masses.

## Data Availability

The original contributions presented in the study are included in the article/Supplementary Material, further inquiries can be directed to the corresponding authors.
