# Supplementary material for: A case report of childhood cutaneous cartilaginous tumor located in the neck
Source: Front Pediatr. 2025 Oct 10;13:1607480. doi: 10.3389/fped.2025.1607480 (PMC12549278; doi:10.3389/fped.2025.1607480)
Supplement: Supplementary file 1 [file Datasheet1.pdf]

患者知情同意书

您好！您在我院接受治疗期间，我们对您的病例进行了详细观察与研究。现拟将您的病例撰写成病例报告发表在医学期刊，旨在为临床医生提供参考，以帮助更多类似疾病的患者。在此过程中，我们将严格遵守医学伦理和相关法律法规，充分保护您的权益。

目的说明

本次撰写病例报告并发表的主要目的在于：分享您独特的疾病表现、诊断过程以及治疗经验，让全球范围内的医学工作者能够从中获取知识，提升对该疾病的认识和治疗水平，从而造福更多患者。

内容披露

病例报告中可能会涉及您的基本信息，如年龄、性别等；疾病相关信息，包括症状、病史、检查结果、诊断结论和治疗过程等。但我们会对您的个人可识别信息，如姓名、身份证号、家庭住址、联系方式等进行严格保密处理，在发表的病例报告中不会直接出现这些信息，仅保留与医学研究相关的必要内容，且会采用化名或编码的形式来指代您，确保您的身份不被泄露。

患者权益保障

您有权自主决定是否同意我们发表您的病例报告。如果您同意，在发表过程中及发表后，若您对病例报告的内容或处理方式有任何疑问或担忧，可随时与我们联系。我们将及时为您解答，并根据您的合理要求进行必要的调整。

若您不同意发表，这绝不会影响您在我院后续的医疗服务质量，我们仍将一如既往地为您提供专业、优质的医疗服务。

保密措施

为保护您的隐私，我们将采取一系列严格的保密措施。除对个人可识别信息进行隐匿处理外，在撰写病例报告相关资料过程中，也将严格限制知晓人员范围，确保资料仅在必要的医疗团队和参与病例报告撰写、审核的相关人员间流通。

患者或法定代理人 / 监护人签字：[illegible]

（若患者为未成年人、无行为能力或限制行为能力人，由法定代理人 / 监护人签字）

与患者关系：[illegible]

日期：2025.3.29
